# Tonsillectomy Under the Microscope: Is Routine Histopathological Analysis Necessary?

**DOI:** 10.7759/cureus.71212

**Published:** 2024-10-10

**Authors:** Emad Al Duhirat, Rani Hammoud, Fatima Emam, Ahmad R Al-Qudimat, Ahmed Shaikh, Abdelkareem Alhyari, Mansour Al Sulaiti, Hamad Al Saey, Shanmugam Ganesan

**Affiliations:** 1 Department of Otolaryngology-Head and Neck Surgery, Hamad Medical Corporation, Doha, QAT; 2 Department of Radiology, Hamad Medical Corporation, Doha, QAT; 3 Department of Public Health, Qatar University, Doha, QAT; 4 Surgical Research Section, Department of Surgery, Hamad Medical Corporation, Doha, QAT; 5 Department of Pathology, Hamad Medical Corporation, Doha, QAT

**Keywords:** histopathology, lymphoma, malignancy, squamous cell carcinoma (scc), tonsillectomy

## Abstract

Introduction

Tonsillectomy is the most common surgery in otorhinolaryngology worldwide, with many indications in adults and children. Traditionally, all tonsillectomy specimens are routinely submitted for histopathological examination, even in the absence of clinical suspicion of malignancy. This practice has been debated due to its resource implications and the low yield of malignancy in routine cases. In this study, we aim to determine the prevalence of occult malignancy in our population and assess the necessity of histopathology examination after routine tonsillectomies.

Methodology

This retrospective chart review was conducted on patients who underwent tonsillectomy between January 2018 and December 2019 at a single tertiary care center. The patients were divided into two groups: those undergoing routine tonsillectomy without clinical suspicion of malignancy and those with suspected malignancy based on preoperative evaluation.

Results

A total of 1857 tonsillectomy cases were analyzed, comprising 1839 routine cases with no suspicion of malignancy and 18 cases where malignancy was suspected. Among the routine cases, 1609 (87.5%) were pediatric patients, and 230 (12.5%) were adult patients. All cases in the suspected malignancy group were adults.

In the routine tonsillectomy group, recurrent tonsillitis was the most common indication for surgery, observed in 821 (51.1%) pediatric patients and 160 (69.6%) adult patients. Reactive lymphoid hyperplasia was the most common histopathological finding, present in 1589 (98.8%) pediatric cases and 187 (81.3%) adult cases. No unexpected malignancies were identified in the routine tonsillectomy group (0%).

In the suspected malignancy group, reactive lymphoid hyperplasia was identified in 13 cases (72.2%), lymphoma in four cases (22.2%), and squamous cell carcinoma (SCC) in one case (5.6%). All patients diagnosed with lymphoma presented with visible tonsillar lesions and palpable neck lymph nodes, three (75%) exhibited tonsillar asymmetry, and two (50%) reported weight loss. The single patient with SCC had asymmetrical tonsils, a visible tonsillar lesion, and palpable neck lymph nodes and experienced weight loss.

Conclusion

The study findings demonstrated no cases of unexpected malignancy in routine tonsillectomy specimens, reinforcing the argument that routine histopathological examination may not be necessary in the absence of clinical suspicion of malignancy. These results, along with considerations of cost-effectiveness and resource optimization, suggest that histopathological analysis may be reserved only for cases with clinical indicators of malignancy.

## Introduction

Tonsillectomy is the most common surgery in otorhinolaryngology worldwide, with hundreds of thousands of procedures each year. Most of the tonsillectomy's indications are benign and nonneoplastic. Indications include obstructive hypertrophy, chronic tonsillitis or recurrent acute tonsillitis, and biopsies in case of suspected malignancy [1,2]. The histopathological examination of tonsillectomy specimens is practiced widely either as an institutional rule or as a physician preference; the reasons are not only to diagnose abnormal tissue but also for its medicolegal purposes. Recently, this practice has been questioned, given the low rate of unexpected occult malignancy in the literature, ranging from 0.01% to 0.015% [3-5]. In addition, this practice significantly utilizes healthcare resources and increases the burden on the healthcare workforce, with estimates suggesting an annual expenditure of approximately $68 million in the United States solely on histopathological examination of tonsillectomy specimens [3,6,7]. The low incidence of tonsillar malignancy without clinical suspicion and the potential savings make this an interesting topic to explore [4,5,8].

In this study, we analyzed the medical charts of patients who underwent a tonsillectomy at our tertiary care center. Our primary aim was to determine the prevalence of occult malignancy in our population and assess the necessity of routine histopathological examination in these cases. Additionally, we sought to determine the malignancy rate among patients with clinical suspicion of tonsillar malignancy.

## Materials and methods

Study design

This retrospective descriptive study reviewed the medical records of all patients who underwent tonsillar surgery between January 2018 and December 2019 at Ambulatory Care Center, Hamad Medical Corporation (HMC). The study has received ethical approval from the HMC Medical Research Center (MRC) (approval number: MRC-01-24-036).

Inclusion criteria

The inclusion criteria are as follows: patients who had undergone a unilateral or bilateral tonsillectomy, regardless of the surgical technique; patients who had concomitant adenoidectomy alongside tonsillectomy; patients who had undergone a unilateral or bilateral tonsillectomy for diagnostic purposes, such as tonsillar biopsy, regardless of the surgical technique; and patients of all ages, with no restrictions on demographic factors.

Exclusion criteria

The exclusion criteria are as follows: patients with a confirmed tonsillar malignancy prior to surgery, patients who had undergone tonsillectomy as part of definitive treatment of known tonsillar malignancy, patients with incomplete or missing medical records, and patients with no histopathological analysis.

Data collection

Demographic and clinical data, including age, gender, nationality, the type of procedure performed, clinical indications for surgery, and histopathology results, were extracted from the medical charts.

In regard to age, patients aged equal to or less than 18 years are considered pediatrics, and those above the age of 18 are considered adults. Regarding nationality, patients are classified into regions according to the World Health Organization.

Patients were categorized into two groups: those with suspected malignancy and those undergoing routine tonsillectomy without suspicion of malignancy (the "routine group"). The suspected malignancy group consisted of patients presenting with concerning clinical features, such as asymmetrical tonsils, the presence of a neck mass or persistent lymphadenopathy, visible tonsillar mass or ulceration, and unexplained weight loss.

Data analysis

Descriptive statistical methods were applied to summarize the demographic, clinical, and histopathological data. Categorical variables are presented as frequencies and percentages, while continuous variables are reported as means with standard deviations (SD). Since the study is descriptive, no inferential statistics or hypothesis testing was conducted.

## Results

General demographics

Of 1891 patient charts screened, 1857 (98.2%) met the inclusion criteria, and 34 (1.8%) were excluded due to missing histopathological results. The mean age of the cohort was 9.36 ± 10.53 years. The male-to-female ratio was 1.34:1, with males comprising 57.1% and females 42.9% of the cases. The study has patients from 46 nationalities; the majority, 1472 (79.27%), are from countries within the Eastern Mediterranean Region (EMR), followed by 170 (9.15%) in the African Region (AFR) and 102 (5.49%) in the Southeast Asia Region (SEAR). Smaller groups came from the Western Pacific Region (WPR), the European Region (EUR), and the Region of the Americas (AMR), which made up 25 (1.35%), 20 (1.08%), and 18 (0.97%), respectively (Table 1).

**Table 1:** Patient demographics WHO: World Health Organization

	Value
Patients Included, N	1857
Mean Age (Years)	9.36 ± 10.53
Pediatrics, N (%)	1609 (86.7%)
Adults, N (%)	248 (13.3%)
Male-to-Female Ratio	1.34:1
Males, N (%)	1060 (57.1%)
Females, N (%)	797 (42.9%)
Region Distribution According to the WHO	
African Region (AFR), N (%)	170 (9.15%)
Region of the Americas (AMR), N (%)	18 (0.97%)
Eastern Mediterranean Region (EMR), N (%)	1472 (79.27%)
European Region (EUR), N (%)	20 (1.08%)
Southeast Asia Region (SEAR), N (%)	102 (5.49%)
Western Pacific Region (WPR), N (%)	25 (1.35%)

Clinical characteristics of the patients in the routine tonsillectomy group

This group comprised 1839 cases, with 1609 (87.5%) pediatric patients and 230 (12.5%) adult patients. The mean age of the pediatric group was 5.67 ± 3.67, while the adult group had a mean age of 32.23 ± 9.58 years. Recurrent tonsillitis was the most common indication for surgery in both groups, present in 821 (51.0%) pediatric cases and 160 (69.6%) adult cases.

Histopathologically, reactive lymphoid hyperplasia was the most common finding, observed in 1589 (98.8%) pediatric cases and 187 (81.3%) adult cases. Reactive lymphoid hyperplasia with actinomycosis was the next most common finding. No malignancies were detected in either the pediatric or the adult routine tonsillectomy groups (Table 2).

**Table 2:** Clinical characteristics of the patients in the routine tonsillectomy group

	Pediatrics	Adults	Total
Number of Cases (N, %)	1609 (87.5%)	230 (12.5%)	1839
Mean Age (Years)	5.67 ± 3.67	32.23 ± 9.58	
Indication of Surgery			
Recurrent Tonsillitis (N, %)	821 (51.03%)	160 (69.57%)	981
Obstructive Sleep-Disordered Breathing (N, %)	336 (20.88%)	65 (28.26%)	401
Obstructive Sleep-Disordered Breathing and Recurrent Tonsillitis (N, %)	452 (28.09%)	5 (2.17%)	457
Type of Surgery			
Adenotonsillectomy (N, %)	1515 (94.16%)	8 (3.48%)	1523
Tonsillectomy (N, %)	94 (5.84%)	222 (96.52%)	316
Histopathology Results			
Reactive Lymphoid Hyperplasia (N, %)	1589 (98.76%)	187 (81.30%)	1776
Reactive Lymphoid Hyperplasia With Actinomycosis (N, %)	20 (1.24%)	42 (18.26%)	62
Reactive Lymphoid Hyperplasia With Lymphangiomatous Polyp (N, %)	0 (0.00%)	1 (0.43%)	1
Malignancy (N, %)	0 (0.00%)	0 (0.00%)	0

Clinical characteristics of the patients in the suspected malignancy group

This group comprised 18 cases, all of which are adults. The mean age of this group was 47.50 ± 13.36 years, with a male-to-female ratio of 2:1. Among these patients, nine (50%) were tobacco users, and two (11.1%) reported alcohol consumption. None of the patients had a family history of oropharyngeal cancer. Clinically, 14 (77.8%) presented with asymmetrical tonsils, seven (38.9%) had visible tonsillar lesions, and six (33.3%) had palpable lymph nodes on physical examination. Nine (50%) underwent diagnostic unilateral tonsillar biopsy, six (33.3%) underwent unilateral tonsillectomy, and the remaining three (16.7%) underwent bilateral tonsillectomy. The histopathology results are distributed as follows: reactive lymphoid hyperplasia, 13 (72.22%); lymphoma, four (22.22%); and squamous cell carcinoma (SCC), one (5.56%)** **(Table 3).

**Table 3:** Clinical characteristics of the patients with suspected tonsillar malignancy Total: N = 18

	Value
Patient Characteristics	
Mean Age (Years)	47.5 ± 13.36
Male, N (%)	12 (66.7%)
Female, N (%)	6 (33.3%)
Tobacco Consumer, N (%)	9 (50%)
Alcohol Consumer, N (%)	2 (11.1%)
Family History of Oropharyngeal Cancer, N (%)	0 (0%)
Asymmetrical Tonsils, N (%)	14 (77.8%)
Tonsillar Lesion/Mass, N (%)	7 (38.9%)
Palpable Neck Mass, N (%)	6 (33.3%)
Unexpected Weight Loss, N (%)	3 (16.7%)
Type of Surgery	
Unilateral Tonsillar Biopsy	9 (50%)
Unilateral Tonsillectomy	6 (33.3%)
Bilateral Tonsillectomy	3 (16.7%)
Histopathology Results	
Lymphoma, N (%)	4 (22.2%)
Squamous Cell Carcinoma, N (%)	1 (5.6%)
Reactive Lymphoid Hyperplasia, N (%)	13 (72.2%)

All patients diagnosed with lymphoma presented with visible tonsillar lesion/mass and palpable neck lymph nodes; three (75%) exhibited tonsillar asymmetry, and two (50%) reported weight loss. The single patient with SCC had asymmetrical tonsils, a visible tonsillar lesion, palpable neck lymph nodes, and experienced weight loss.

## Discussion

Histopathological analysis is a meticulous process. The analyzed tissue undergoes several sequential steps to ensure optimal preservation and preparation for microscopic examination. These include tissue fixation, dehydration, and embedding, which are costly and time-consuming. To avoid potentially unnecessary interventions and investigations, physicians must prioritize practicality in effectively utilizing healthcare resources and funds to reduce the financial burden on the healthcare system and potentially enhance patient satisfaction [9].

The most common pathological conditions that can affect the tonsils are recurrent and chronic infections. At a microscopic level, these chronically and repeatedly infected tonsils constantly appear as follicular lymphoid hyperplasia with surrounding inflammatory cells, and in some cases, fibrosis can be seen [6]. Although the majority of tonsil specimens will show only hyperplasia, tonsils are not an uncommon site for primary head and neck neoplasms [6]. Twenty-five percent of tonsillar neoplasms are benign, such as squamous cell papilloma and lymphangiomas. Regarding malignant neoplasms, SCC is the most common, compromising around 85%, followed by lymphoma (16%) and other lymphoepithelial carcinomas (3%-7%) [6,10].

Some have advocated for a routine histopathological examination of all tonsillectomy specimens to rule out occult malignancy. Over 13 years, Ridgway et al. reported six pediatric cases diagnosed with non-Hodgkin's lymphoma from routine tonsillectomy specimens, suggesting the need for pathological examination [11]. However, a review of these cases revealed that all had one or more preoperative clinical risk factors, which should have prompted suspicion [6,11]. The rate of unexpected tonsillar malignancy after routine tonsillectomy is as low as 0.01%-0.015% [3-5]. Despite the rarity of tonsillar malignancies, they remain a serious threat, highlighting the need for an efficient predictive model to identify high-risk individuals. History-taking and physical examination are fundamental in identifying cases that warrant closer scrutiny for potential malignancy in tonsillectomy patients. Findings such as asymmetrical tonsils, visible tonsillar lesions or masses, palpable cervical lymph nodes, history of cancer, and unexpected weight loss may suggest tonsillar malignancy. Beaty et al. termed these as "high-risk factors" associated with tonsil malignancies. In their study, they compared 428 patients with benign preoperative diagnoses to 25 patients with malignancies diagnosed post-tonsillectomy; they found that patients without these risk factors had no malignancies [12].

Our study shed light on the prevalence of occult malignancy in routine tonsillectomy cases within our region. The noteworthy observation in our study is the absence of unexpected malignancy among the 1839 patients who underwent routine tonsillectomy. Tonsillar malignancy was seen in five out of the 18 cases who had preoperative risk factors for tonsillar neoplasm. These findings align with the recent existing literature reporting low rates of occult malignancy elsewhere in the world [3-5,13,14]. Subsequently, this raises questions about the necessity of the routine histopathology examination of tonsils in the absence of clinical suspicion of malignancy. This practice, a physician's personal choice or an institutional regulation, has significant implications on the cost, patient satisfaction levels, time, and resources used by pathologists and technicians, ultimately affecting the overall efficiency of healthcare system delivery within institutions [15].

The cost of routine histopathological examination is estimated to range from $35 to $69 per tonsil, which is comparable to the costs incurred at our institution [6,10,16]. This financial burden translates to an annual expenditure of approximately $64365-$126891 at our institution, a cost that could potentially be allocated elsewhere. Furthermore, given the high number of daily tonsillectomies, routine histological examination increases the workload pressures on pathologists. This practice can lead to backlogs and delays in reporting, potentially hindering the timely management of urgent and malignant cases [17].

Study limitation

Due to the retrospective design, the potential for selection bias, and the specific demographics and healthcare practices prevalent in Qatar and the region, our results are limited in generalizability to other populations.

## Conclusions

Our study's findings revealed no unexpected malignancies in the specimens of the 1839 patients who underwent routine tonsillectomies. Tonsillar malignancy was only observed in the suspected malignancy group. Our findings align with the growing body of evidence suggesting that routine histopathological examination may not be required in every patient, while it is encouraged in patients suspected clinically to have unusual preoperative findings or risk factors.
